# Treatment of Traumatic Tetanus

**Published:** 1854-02

**Authors:** Freeman Knowles

**Affiliations:** Professor of Theory and Practice of Medicine in the Iowa Medical College


### IOWA MEDICAL JOURNAL.

VOL. I. KEOKUK, IOWA, FEBRUARY, 1854. NO 7.

### ORIGINAL COMMUNICATIONS.

### *Successful Treatment of Traumatic Tetanus.*

BY FREEMAN KNOWLES, M. D.

Professor of Theory and Practice of Medicine in the Iowa Medical College.

There is no malady, perhaps, to which the human frame is subject, that has proved more perplexing to the Physician than Traumatic Tetanus; and none that has thus far more completely in most cases baffled all the efforts at successful treatment which the most intelligent and skillful have been able to adopt. If we are to judge of its fatality in the reports of the best systematic writers, we must lay it down as among the *opprobia medicorum* of our day, as well as those of our predecessors.

While the special pathology of this affection seems to be better understood than any general principle which is to guide us in our treatment, some refering the affection to the spinal chord and others to the brain or ganglionic centers, none tell us whether it gets there by reflex action of the nervous system or is conveyed by continuous inflammation of a nervous trunk; or whether it is the result of general excitement or irritation of the nervous system, producing local determination to the peculiar part where the lesion may be found, in the same manner that we have local inflammation frequently arising as the consequence of idiopathic fever.

For may own part, I have little doubt that the latter is the fact.

When we reflect that idiopathic tetanus is comparatively a rare disease and after death occasionally eludes all traces of its seat or point of radition when no local lesion exists—is it not fair to suppose that death may result from such a state of the nervous system independent of any radiatory point except the one which superinduced this peculiar condition of the nervous system? While one class of pathologists consider it essentially a hyperœmic disease and use the lancet as the Hercules that is to relax the over strained muscles and give back the control to the will, others consider it an anœmic disease and recommend wine, bark, carbonate of iron, mercury and opium, prussic acid, belladonna, musk and digitalis, oil of turpentine, strycnia and tobacco.

Under this whole array of powerful drugs the mortality is certainly lamentable, and little better now than in the days of Hippocrates; for he tells us that "Tetanus which supervenes upon a wound is mortal." Henen declares that he never knew a case of acute traumatic tetanus to recover, and Dr. Dickson found all curative means followed by unqualified disappointment. The quantity of certain drugs which have been borne in this fearful malady is truly surprising particularly those which effect the nervous system. Opium has for instance been taken to the amount of an ounce of the solid gum, in divided doses, every 24 hours, for twenty-two days, (*London Med. and Chirurg. Transactions. Vol. 2.*) and the amount of some other drugs that has been used, is equally remarkable.

I have sometimes asked myself, if some of the deaths have not resulted from such fearful doses of poisonous medicines. Dr. Abernethy says he has found opium enough in the stomach, after death, to poision ten persons. Query—how much had the opium to do with the death? Sir James McGregor mentions a case that was relieved by tobacco, but ultimately sunk from the effects of the remedy, and this seems to be about the same case, with the use of digilatis. Considering the cumulative character of the remedy. is it too much to infer, that when the remedy has been used to the extent by which the spasm is relieved, it may then exert its peculiar power upon the heart and still its pulsations forever. Chloroform is a more recent remedy and is capable unquestionably of being employed to great advantage in the modification of the most alarming symptoms, holding the disease in check until the pathological condition on which the disease depends, has been obviated. How far it may operate as a remedy for the permanent cure of the affection, is very much in doubt, and requires much more careful and varied experiment, before its true place and character in the list of therapeutic agents for the cure of tetanus, becomes fixed.

From the foregoing considerations, it becomes very obvious that as yet we have no system of practice, based upon any general principles, that serves as a land-mark or guiding star, to direct the practitioner in his treatment of this fearful malady. In this state of uncertainty and doubt, we shall perhaps be pardoned for obtruding our own opinions and a new remedy upon the notice of the profession, asking only just that amount of consideration for it, that its merits demand.

Having for some years been in the habit of using Lobelia Inflata, in several spasmodic affections, with the most marked advantage, I was induced to make a fair trial of the article in the treatment of tetanus; and to my inexpressible gratification it has achieved in the cases in which I have used it, all that I could ask. Still I am fully aware, that in order to a full understanding of its merits, a much wider range of experience is demanded. For that purpose it is now submitted to the scrutiny of those who are seeking after truth, and every means by which suffering humanity may be alleviated.

Case 1. Mrs. T. aged 35,nervous temperament, spare made, enjoying good health, occupation a seamstress, on the 10th of July accidentally drove her needle under the thumb nail of the left hand, about one fourth of an inch. That night the thumb became hot and painful, and on the fourth day the nail had become loosened from the integuments, except at the root, and course unhealthy granulations were turned out, a line in width, all round three sides ot the nail. On the night of the 14th, without any premonitory warning, violent pain was felt in the left shoulder, extending to the neck and ultimately to the lower jaw; in about an hour from the time her jaws became affected, spasms were felt along the back, arms, lower extremities and abdomen. I saw the patient about 12 o'clock that night, two hours after the attack, she residing in the country, five miles distant. I found her in violent spasms, her head and heels drawn backward, forming a crescent,all the voluntary muscles more or less participating in the violence of the affection; slight remissions occurring about ten minutes;apart she complained of the most horrible agony,during the paroxyisms; deglutition was entirely suspended; patient complained of a sensation about the jaws as if the teeth were being driven through them; pulse for most of the time, feeble and irregular; mind unclouded. Gave the patient the following prescription :

Tict. Opii ℥jj.

Spts. Camphor.

Sul. Ether, a a ℥ss.

M.

Dose, about a drachm between each paroxyism, till three fourths of the quantity was used. I then reflected if the spasms should suddenly cease, my patient would probably die from the effect of the narcotic. After waiting about an hour with no abatement of the violence of the symptoms, I resolved to try the relaxing effect of the tincture of lobelia, and accordingly at the first period the patient could swallow, gave her a full table spoonful of a saturated tinct. of lobelia made from the seed. In about twenty minutes there was a manifest mitigation in the violence of the paroxyism. I then in the hope of producing emesis, administered another table-spoonful, but without producing any change, only a gradual relaxation of the spasm. In this state fearing the consequences of one and a half ounces of laudanum, I determined to produce emesis if possible, and for that purpose gave a decoction of capsicum in table spoonful doses, as often as the patient could swallow. After the third spoonful very active emesis took place, and with it every vestige of the spasm disappeared. I continued the patient under the influence of the remedy in small but repeated doses for about twenty-four hours and then discontinued it. Four days after I amputated the thumb; the wound healed kindly with no return of any of the spasmodic symptoms.

I have detailed the case above, because it was the first case in which this remedy was used, and consequently noted at the time. In two other cases, since that time, I have used the same remedy from the beginning, with equal success. The one was a man, 35 years of age, nervo-sanguine temperament, and the other a lad of 17, of nervous billious temperament. The former was caused by the extraction of a carious tooth, which had for a long time been the seat of neuralgia, and the latter was caused by steping upon a rusty nail, which passed into the heel about three fourths of an inch. In both of the above cases, tincture of lobelia was given in drachm doses about every ten minutes, till mitigation of the symptoms became apparent, and then decoction of capsicum, to excite the stomach to emesis. In all the above cases, the violence of *all* the symptoms subsided after free emesis and continuing the remedy, in a few hours all spasmodic indications disappeared.

I should have remarked thath in the case of the boy, I laid open with a scalpel, the wound made by the nail, to the bottom, and encouraged the hemorrhage by cloths rung out of warm water.

I thus submit the above cases to the profession, without comment, hoping that others may find in this potent article as satisfactory results as myself.

